# Age-Correction of Test Scores Reduces the Validity of Mild Cognitive Impairment in Predicting Progression to Dementia

**DOI:** 10.1371/journal.pone.0106284

**Published:** 2014-08-29

**Authors:** Johannes Hessler, Oliver Tucha, Hans Förstl, Edelgard Mösch, Horst Bickel

**Affiliations:** 1 Department of Psychiatry and Psychotherapy, Technische Universität München, Klinikum rechts der Isar, Munich, Germany; 2 Department of Clinical and Developmental Neuropsychology, University of Groningen, Groningen, The Netherlands; University of Manchester, United Kingdom

## Abstract

**Objectives:**

A phase of mild cognitive impairment (MCI) precedes most forms of neurodegenerative dementia. Many definitions of MCI recommend the use of test norms to diagnose cognitive impairment. It is, however, unclear whether the use of norms actually improves the detection of individuals at risk of dementia. Therefore, the effects of age- and education-norms on the validity of test scores in predicting progression to dementia were investigated.

**Methods:**

Baseline cognitive test scores (Syndrome Short Test) of dementia-free participants aged ≥65 were used to predict progression to dementia within three years. Participants were comprehensively examined one, two, and three years after baseline. Test scores were calculated with correction for (1) age and education, (2) education only, (3) age only and (4) without correction. Predictive validity was estimated with Cox proportional hazard regressions. Areas under the curve (AUCs) were calculated for the one-, two-, and three-year intervals.

**Results:**

82 (15.3%) of initially 537 participants, developed dementia. Model coefficients, hazard ratios, and AUCs of all scores were significant (*p*<0.001). Predictive validity was the lowest with age-corrected scores (−2 log likelihood  = 840.90, model fit χ^2^ (1)  = 144.27, HR  = 1.33, AUCs between 0.73 and 0.87) and the highest with education-corrected scores (−2 log likelihood  = 815.80, model fit χ^2^ (1)  = 171.16, HR  = 1.34, AUCs between 0.85 and 0.88).

**Conclusion:**

The predictive validity of test scores is markedly reduced by age-correction. Therefore, definitions of MCI should not recommend the use of age-norms in order to improve the detection of individuals at risk of dementia.

## Introduction

Many attempts have been made to describe and define the gray area of mild cognitive impairment (MCI) that lies between healthy aging and dementia [1], [2]. The hallmark of all definitions of MCI is an objective impairment of memory or multiple cognitive domains. Yet, most approaches only provide ambiguous recommendations for diagnostic procedures and operationalization of criteria. For example, cognitive impairment in MCI has been defined as test performance below expectations for age and education [3], [4] or, more decidedly, as a score lower than 1 to 2 standard deviations (*SD*s) below the mean of young adults [5], the age-matched group [6], or the age- and education-matched group [7], [8].

Despite this operational elusiveness, the presumed clinical value of MCI is its ability to identify individuals that are at higher risk of dementia [9]. Mild cognitive deficits usually emerge several years prior to a clinical diagnosis of dementia [10] and higher rates of progression to dementia have been associated with a diagnosis of MCI [4], [11]–[13]. Importantly, the estimates showed considerable heterogeneity across studies and the most common outcome was remission to normal cognitive functioning with a rate of around 40% [14], [15]. Recent findings indicate that some of the remitted cases may remain at higher risk of progressing to dementia [16]. Even though substantial controversies remain about the nature of MCI and its association with dementia [17], it is reasonable to assume that, at least for a subgroup of individuals, MCI is closely connected to the risk of progressing to dementia.

It has been argued that ambiguous definitions of MCI informed the inconsistent operationalization and implementation of the diagnostic criteria of MCI in several studies [18], [19]. As a consequence, equivocal findings on the association between MCI and dementia may be partly due to artifacts introduced by this heterogeneity [13], [20]. Addressing and resolving these issues step by step may increase the predictive validity and, thereby, the clinical value of MCI, as individuals at higher risk of developing dementia could be identified with greater accuracy [20]. In their review, Bruscoli and Lovestone [11] found in all included studies that baseline cognitive performance was the best predictor of conversion from MCI to dementia. Cognitive test outcomes may, therefore, represent a promising toehold for improving the predictability of progression from MCI to dementia. The diagnosis of cognitive impairment due to “performance below expectations for age and education” has been identified as a common source of variation across studies [18], [20] and considered problematic in the context of MCI [21]. There is evidence from cross-sectional studies suggesting that correcting test scores for risk factors like age, gender, and education decreases their sensitivity in detecting dementia [22]–[27]. To our knowledge, however, this notion has neither been examined in a prospective longitudinal study nor with regard to MCI. Therefore, the present study investigated the effect of age- and education-norms on the validity of test scores in predicting progression to dementia.

As the incidence of dementia increases exponentially after age 65 [28], the risk of developing dementia increases with each year. Age-norms as they are commonly used, however, become more forgiving with higher age. They tolerate more errors and classify individuals with lower performance as still within normal range and, therefore, at lower risk of dementia. Applying a cut-off at 2 *SD*s below the mean of an age-matched group, for example, classifies around 2.3% of the cases as being at risk of dementia. As the risk of dementia exceeds 2.3% in higher age groups [28], risk estimation becomes increasingly inaccurate and more cases are missed. Actually, individuals with higher age (high risk group) had to show better performance than younger individuals (low risk group) in order to be considered at lower risk, since better performance at higher age would indicate higher brain reserve [29]. Consequently, it has been argued that the application of age-norms underestimates the risk of dementia in higher age groups [21].

Education is thought to moderate the association between age and dementia. Higher levels of education are considered to reflect increased cognitive reserve, which can delay the emergence of symptoms of dementia despite progressing neuropathology [29]–[31]. The application of education-norms accommodates this assumption. If an individual with higher education achieves test scores similar to an individual with lower education, the former might have lost the advantage of the protective effect due to an already incipient cognitive decline and is, therefore, at higher risk of progressing to dementia. This means that in the absence of cognitive impairment, the more educated person would be expected to score higher than the less educated person in order to be considered “normal”. Evaluating test performance of individuals with different levels of education with the same standard would neglect this difference and likely underestimate the risk of dementia for higher educated and overestimate the risk for lower educated.

Another way of thinking about age and education within the context of test performance and risk of dementia is as confounding variables. Age and educational level are associated with both test performance and the risk of dementia and may partly account for the relationship between the latter. In order to increase the predictive validity of cognitive test scores, it would be favorable to evaluate cognitive test performance in a way that accurately reflects an individual's risk of dementia regardless of their age and education [24]. More technically put, test scores should still have high predictive validity when age and education are controlled for.

As age- or age- and education-norms are commonly used to diagnose cognitive impairment in MCI [18], [20], previous conversion studies might have misjudged the risk of dementia in some of their participants. Therefore, the aim of the present study was to investigate how the application of age- and education-norms in cognitive performance evaluation affects the validity of test scores in predicting progression to dementia. Knowledge about the implications of norms allows for improving the clinical value of MCI, which is identifying those individuals who are at an increased risk of progressing to dementia.

In the present study, new cases of dementia within three years were predicted from baseline neuropsychological test performance that was evaluated with correcting for age, education, age and education, or with no correction. Based on the rationale that the estimation of dementia risk is impaired by age-correction of test scores and improved by education-correction, we hypothesize that (1) education-corrected scores have the highest validity in predicting progression to dementia, whereas age-corrected scores have the lowest predictive validity and that (2) education-corrected scores reduce the predictive validity of the confounders age and education the most.

## Method

### Participants

Participants were older patients of three general hospitals in Munich, Germany. Inclusion criteria were age between 65 and 85 and residence in the larger area of the city. Exclusion criteria were severe physical illness; manifest dementia; residence in a nursing home; need for care according to the criteria of the German long-term care insurance plan; blindness or deafness; insufficient proficiency in German; and imminent release from the hospital within 48 hours. More details about the sample and the screening in the hospitals that are not directly related to the present study are published [32].

### Study protocol and materials

The study protocol was approved by the institutional review board of the Faculty of Medicine at the Technische Universität München. For the purpose of the present study, data of patients with initially no or only mild cognitive impairment was used. Therefore, all participants were considered capable of giving informed consent and written consent was obtained from all participants. Even in the case of more pronounced cognitive impairment, prevailing German legal norms only require consent of a third party when they have been previously appointed as legal guardians. As for none of the participants a legal guardianship was established, no surrogate consent had to be obtained.

The patients were examined at five points in time by trained psychiatrists and psychologists. An initial screening was conducted in the hospitals (T0), when all participants had inpatient status. Approximately 3 months after the screening, when all participants were discharged from the hospital, they were visited at their homes for a first follow-up (T1). Three further follow-ups (T2, T3, and T4) took place 1, 2, and 3 years after T1.

For the purpose of our study, only data collected at the four follow-ups were used. Therefore, T1 served as the baseline and T2, T3, and T4 as follow-up examinations. The circumstances for a thorough neuropsychological assessment are not optimal in a hospital setting, as tests may have to be administered at the bedside, interruptions are likely to occur, and standardized test conduction cannot be guaranteed. At T1, the patients were visited at their homes, which allowed for more comprehensive and standardized assessment of cognitive functioning. Also, the screening and the follow-ups partly differed in the employed test battery. The Syndrome Short Test (Syndrom Kurztest, SKT) [33], which we chose as the instrument to examine our research question with (for reasons stated below), was not administered in the hospital but at T1 to T4. This was done because the SKT requires the test-taker to handle different materials (e.g., magnets) and also includes timed tests, which makes bedside testing difficult.

Dementia at the screening in the hospital was diagnosed with the Structured Interview for the Diagnosis of Dementia of the Alzheimer Type, Multi-Infarct Type, and Dementia of other Etiology according to DSM-III-R, DSM-IV and ICD-10 [34]. Its core instrument is a test battery consisting of 55 items, including the 30 items of the Mini Mental State Examination (MMSE) [35]. The test battery of the follow-ups (T1–T4) is described in the following. Cognitive performance was assessed by means of the MMSE, a clock drawing test [36], a verbal fluency test (number of animals within 60 seconds), and the SKT. First published in the seventies [37], the SKT is a validated and internationally used test [38] that has been shown to be sensitive to cognitive impairment in MCI and dementia [39] and correlate well with other established measures, as the MMSE and the clock test [40]. The SKT is especially suited for the purpose of the present study and was, therefore, chosen as main instrument to examine the present research question with. First, its scoring procedure explicitly takes the participants' age and educational level into account. Second, practice effects in a longitudinal design are reduced due to the availability of five parallel versions. Third, the SKT has been recommended for the assessment of attention and memory in MCI and mild dementia [39].

The SKT consists of nine subtests, three loading on memory and six on attention. The attention tasks have to be completed within 60 seconds. The memory tasks are scored as number of errors committed and the attention tasks as seconds needed to complete the tasks. As described in the manual, calculating the SKT total score requires the transformation of error- and time-scores into normed scores [41]. Norms for six age-groups (17–44; 45–54; 55–64; 65–74; 75–84; ≥85) and three levels of estimated premorbid IQ (<90; 90–110; >110) are provided in the manual. According to the test author's instruction, premorbid IQ was estimated by educational level [41]. The following classification was used for the purpose of the present study: individuals with no formal education were allocated to the below average IQ group (<90), primary compulsory school graduates to the average group (90–110), and graduates of higher schools to the above average group (>110). For each subtest 0–3 points are given with higher scores indicating higher levels of impairment. Sum scores can be calculated for the cognitive domains separately (0–9 for memory and 0–18 for attention) or combined (0–27). For an example of the transformation from raw into normed scores, an individual aged 74 and with a low level of education is considered. She commits 6 errors on the first memory subtask. Based on age and estimated IQ, the raw score of 6 is transformed into a normed score of 0, indicating no deficits. For an individual aged 64 with a high level of education, the raw score of 6 is transformed into a normed score of 1, indicating slight deficits. This example illustrates that the SKT's norms allow older and less educated individuals to perform less well on the subtasks and still be regarded as unimpaired. Younger and higher educated individuals, however, have to perform better in order to be regarded as unimpaired.

Subjective memory impairment was assessed with items of the Cambridge Examination for Mental Disorders of the Elderly [42]. The participants' functional level of daily activities was established in interviews with knowledgeable informants by using the Bayer Activities of Daily Living Scale [43] and the Informant Questionnaire on Cognitive Decline in the Elderly [44]. Depressive symptoms were assessed with the 15-item version of the Geriatric Depression Scale [45].

Based on all the above information, each participant's cognitive status was rated on the Clinical Dementia Rating Scale (CDR) [46]. The CDR discriminates between five stages of cognitive impairment (with corresponding numerical indices): none (0), very mild (0.5), mild (1), moderate (2), and severe (3). For the analysis only data of participants with no or mild cognitive impairment (CDR  = 0 or 0.5, respectively) at T1 were included in order to predict progression to dementia. Consequently, participants with mild, moderate, and severe dementia (CDR  = 1, 2, and 3, respectively) at T1 were excluded. Incident dementia at T2, T3, and T4 was diagnosed according to the DSM-IV criteria and operationalized as CDR ≥1 with a previous CDR  = 0 or 0.5 at T1.

### Age- and education-correction of SKT scores

Because the aim of our study was to map the change in predictive validity of neuropsychological test scores when age- and education-norms are considered in the scoring procedure, SKT total scores were calculated according to four different procedures.

(1) SKT_Corrected_: Application of a differentiated transformation procedure taking individual age-group membership (65–74; 75–84; ≥85) and educational level (no education; primary compulsory school; higher schools) into account. This is the standard scoring procedure of the SKT [41] and the derived scores are age- and education-corrected.

(2) SKT_Uncorrected_: Application of an average transformation procedure with age 65–74 and primary compulsory school education, regardless of the participants' age and educational level. The derived scores are age- and education-uncorrected. As the SKT assesses attention and memory with different procedures, raw error and time scores have to be transformed into scores between 0 and 3 to allow for calculating a combined sum score. The calculation of uncorrected scores is characterized by comparing the performance of all participants to the same standard so that no systematic difference between the participants is introduced. This requirement is met with an average transformation procedure.

(3) SKT_Education_: Application of a transformation procedure with average age (65–74) for all participants and a differentiated education according to the individual level. The derived scores are education-corrected and age-uncorrected.

(4) SKT_Age_: Application of a transformation procedure with average education (primary compulsory school) for all participants and a differentiated age-group membership according to the individuals' age. The derived scores are age-corrected and education-uncorrected.

### Statistical analysis

Cox proportional hazard regressions were employed to determine the relative risk of new dementia within three years from baseline (T1) SKT total scores. The dependent variable was defined as new dementia between T2 and T4 or no dementia over the course of the study. For new dementia cases, the time variable was defined by the months between the date of T1 and the date of the follow-up at which dementia was diagnosed for the first time. For dementia-free cases, the time variable was defined by the time in months between T1 and the date of drop-out (e.g., due to death) or study end. Four Cox-regressions were performed using SKT_Corrected_, SKT_Uncorrected_, SKT_Education_, and SKT_Age_ at T1 as respective predictors. Additional Cox-regressions were performed using SKT_Corrected_, SKT_Uncorrected_, SKT_Education_, and SKT_Age_ as predictors and age (in years) alone, as well as age and education (total years of school and occupational training) together as covariates (as described by Sliwinski et al. [23]). This allows for examining to what degree cognitive test scores are independent of the confounding risk factors age and education in their predictive validity [24]. Relative risk of conversion to dementia as predicted by SKT total scores was determined by hazard ratios (HRs) and their 95% confidence intervals (CIs). Model fit was determined by −2 log likelihood as an indicator of variance unaccounted for by SKT total scores and χ^2^-tests for overall fit of the model and improvement over the null-model (i.e., with no predictors). The predictive validity of age and education was described by the regression coefficient *B*, its standard error, the Wald-statistic testing the significance of the HRs, as well as HRs and the respective 95% CIs.

In order to render the relative risks comparable across the four approaches, HRs were weighted for the *SD*s of the respective score. For example, the regression coefficient *B* for SKT_Corrected_ in the Cox-regression ( =  the natural logarithm of the HR of SKT_Corrected_) was multiplied with the *SD* of SKT_Corrected_ at T1. The weighted HR (HR_W_) was then calculated by applying the product to the power of e, that is, e*^B^* ^×*SD*^. For direct comparison, a Cox-regression using a backward selection method (likelihood ratio) was performed. SKT_Corrected_, SKT_Uncorrected_, SKT_Education_, and SKT_Age_ were simultaneously entered as predictors. This method starts with the full model and tests for each predictor whether its removal causes a significant decrease in predictive power as indicated by loss in model χ^2^.

Receiver-operator-characteristics (ROCs) were calculated for a quantitative comparison of the predictive validity of SKT scores. Areas under the curve (AUCs) and their 95% confidence intervals were calculated with SKT_Corrected_, SKT_Uncorrected_, SKT_Education_, and SKT_Age_ at T1 as test variables and new dementia at a specific follow-up as status variable. This was done separately for T2, T3, and T4. This means that for each approach AUCs were calculated for the one-year interval T1–T2, the two-year interval T1–T3, and the three-year interval T1–T4.

Statistical analyses were carried out with SPSS version 20 for Macintosh. The level of significance was set at α≤0.05. As the results of the present study are mostly descriptive, no α-adjustment for multiple hypothesis testing was applied.

## Results

At T1, 562 participants were examined. Sixteen were excluded due to a CDR-rating ≥1 indicating dementia and 9 did not complete the SKT. Consequently, 537 participants (321 female, 59.8%) were included in the analysis. Their mean age at T1 was 75.61 years (*SD*  = 5.47, median  = 75.87). Two-hundred and nine participants (38.9%) were aged between 65 and 74, 316 (58.9%) between 75 and 84, and 12 (2.2%) 85 or older. Three-hundred and thirty (61.5%) graduated from primary compulsory school and 207 (38.5%) graduated from higher schools. There were no participants without formal education in our sample. Mean years of school and occupational training was 9.6 (*SD*  = 2.9, median  = 8). The mean time of participation in the study was 33.8 (*SD*  = 9.8) months. In total, 82 (15.3%) individuals developed new dementia over the course of the study. Mean scores were SKT_Corrected_ = 3.01 (*SD*  = 3.05), SKT_Uncorrected_  = 3.49 (*SD*  = 3.35), SKT_Education_ = 3.93 (*SD*  = 3.48), and SKT_Age_ = 2.66 (*SD*  = 2.97). Details with regard to sample sizes, drop-outs, and new cases of dementia over the course of the study are displayed in Table 1.

**Table 1:** Study summary.

examination	no dementia	new dementia	previously diagnosed with dementia	drop-outs		
				refused	dead	not possible/others
T1 (*N* = 537)	537	*	-	-	-	-
T2 (*N* = 453)	414	39	-	44	32	8
T3 (*N* = 400)	344	27	29	17	33	3
T4 (*N* = 330)	275	16	39	22	36	12

*Note*. * cases with dementia excluded at T1 (*N* = 16).

Table 2 shows the results of the Cox proportional hazard regressions with SKT_Corrected_, SKT_Uncorrected_, SKT_Education_, and SKT_Age_ as respective predictors. For all approaches the model fit coefficients and HRs were significant (all *p*<0.001) and the HRs' CIs small. As can be seen in Table 2, SKT_Uncorrected_ adjusted for age and education had the best predictive validity as determined by model fit statistics. Of the unadjusted models, SKT_Uncorrected_ and SKT_Education_ revealed the most favorable results. SKT_Age_ had the worst model fit. HRs and corresponding 95% CIs were almost similar for all models. SKT_Education_ had the largest HR_W_, SKT_Age_ the smallest.

**Table 2:** Validity of predicting new dementia within three years from adjusted and unadjusted SKT scores at T1: results of the Cox proportional hazard regressions.

SKT total score at T1	Model fit			Relative risk^#^		
	−2 log likelihood	Overall model fit χ^2^ (*df*)	Over null model χ^2^ (*df*)	HR	95% CI	HR_W_
SKT_Corrected_	837.56	145.03 (1)*	94.71 (1)*	1.34*	1.27–1.41	2.44
Age	808.16	172.45 (2)*	124.11 (2)*	1.35*	1.27–1.42	2.44
Age, education	807.84	173.25 (3)*	124.43 (3)*	1.35*	1.28–1.42	2.44
SKT_Uncorrected_	816.07	174.19 (1)*	116.20 (1)*	1.35*	1.29–1.42	2.73
Age	800.61	182.68 (2)*	131.66 (2)*	1.34*	1.27–1.41	2.66
Age, education	797.81	188.70 (3)*	134.46 (3)*	1.34*	1.28–1.42	2.66
SKT_Education_	815.80	171.16 (1)*	116.47 (1)*	1.34*	1.28–1.41	2.77
Age	803.87	178.29 (2)*	128.40 (2)*	1.32*	1.25–1.39	2.63
Age, education	803.76	178.59 (3)*	128.50 (3)*	1.32*	1.25–1.40	2.63
SKT_Age_	840.90	144.27 (1)*	91.37 (1)*	1.33*	1.27–1.40	2.33
Age	810.96	173.25 (2)*	121.31 (2)*	1.34*	1.27–1.42	2.39
Age, education	808.54	179.57 (3)*	123.73 (3)*	1.34*	1.28–1.42	2.39

*Note*. * significant at *p*<0.001: ^#^ HR, 95% CI, and HR_W_ pertain to the respective adjusted or unadjusted SKT score. HR_W_  =  weighted HR; SKT_Corrected_  =  differentiated age and education; SKT_Uncorrected_  =  average age and education; SKT_Education_  =  differentiated education and average age; SKT_Age_  =  differentiated age and average education.

A Cox-regression using a backward selection method included SKT_Uncorrected_ as the only significant predictor (loss in χ^2^ = 116.20, *df* = 1, *p*<0.001). Removal of SKT_Corrected_ (loss in χ^2^ = 0.19, *df* = 1, *p* = 0.663), SKT_Education_ (loss in χ^2^ = 2.03, *df* = 1, *p* = 0.155), and SKT_Age_ (loss in χ^2^ = 3.40, *df* = 1, *p* = 0.065) did not significantly decrease the models predictive power. Table 3 shows the AUCs and the respective 95% CIs for SKT total scores at T1 in predicting new dementia at T2, T3, and T4. Table 4 displays the predictive validity of age and education in predicting new dementia when entered simultaneously with SKT total scores at T1 in a Cox proportional hazard regression.

**Table 3:** Validity of predicting new dementia within one, two, and three years from SKT scores at T1: results of the ROC-analysis.

SKT total score at T1	T2 (*N* = 453)		T3 (*N* = 371)		T4 (*N* = 291)	
	AUC	95% CI	AUC	95% CI	AUC	95% CI
SKT_Corrected_	0.86*	0.81–0.92	0.75*	0.66–0.84	0.78*	0.64–0.91
SKT_Uncorrected_	0.89*	0.84–0.93	0.82*	0.74–0.89	0.82*	0.72–0.92
SKT_Education_	0.88*	0.83–0.93	0.82*	0.75–0.89	0.85*	0.76–0.94
SKT_Age_	0.87*	0.82–0.91	0.76*	0.67–0.85	0.73*	0.57–0.89

*Note*. * asymptotically significant at *p*<0.001. Numbers of new cases of dementia at each examination are shown in Table 1. Cases with dementia at a previous examination were excluded. SKT_Corrected_  =  differentiated age and education; SKT_Uncorrected_  =  average age and education; SKT_Education_  =  differentiated education and average age; SKT_Age_  =  differentiated age and average education.

**Table 4:** Predictive validity of age and education: results of the Cox proportional hazard regressions.

Predictor	*B* (*SE*)	Wald (*df*)	*p*	HR	95% CI HR
SKT_Corrected_					
Age	0.12 (0.02)	27.38 (1)	<0.001	1.13	1.08–1.18
Education	0.02 (0.04)	0.17 (1)	0.682	1.02	0.94–1.09
SKT_Uncorrected_					
Age	0.09 (0.02)	13.97 (1)	<0.001	1.09	1.04–1.14
Education	0.06 (0.04)	3.05 (1)	0.081	1.07	0.99–1.14
SKT_Education_					
Age	0.08 (0.02)	12.09 (1)	0.001	1.09	1.04–1.14
Education	−0.12 (0.04)	0.09 (1)	0.760	0.98	0.92–1.07
SKT_Age_					
Age	0.12 (0.02)	27.01 (1)	<0.001	1.12	1.08–1.17
Education	0.06 (0.04)	2.38 (1)	0.123	1.06	0.96–1.14

*Note*. SKT_Corrected_  =  differentiated age and education; SKT_Uncorrected_  =  average age and education; SKT_Education_  =  differentiated education and average age; SKT_Age_  =  differentiated age and average education.

## Discussion

As many definitions of MCI recommend the use of age- and education-norms [20], we compared the validity of corrected and uncorrected test scores to predict progression to dementia.

We hypothesized that (1) the predictive validity of test scores is increased by education-correction and decreased by age-correction and that (2) education-corrected test scores reduce the predictive validity of the confounders age and education the most. Test scores were calculated according to four procedures (SKT_Corrected_  =  age- and education-corrected; SKT_Uncorrected_  =  uncorrected; SKT_Education_  =  education-corrected and age-uncorrected; and SKT_Age_  =  age-corrected and education-uncorrected) and separately employed as predictors for conversion to dementia within three years. Both corrected and uncorrected scores were highly significant predictors of progression to dementia, even when adjusted for age and education. As hypothesized (1), age- and age- and education corrected scores had lower predictive validity than education-corrected and uncorrected scores, which showed comparable predictive power. In a direct comparison, only uncorrected scores were included in the predictive model, however, the descriptive statistics for education-corrected scores indicated slightly higher predictive accuracy. Confirming our hypothesis (2), education-corrected scores reduced the predictive influence of age and education the most. Though, the advantage over uncorrected scores was only small. As previously described, it is desirable to have test procedures that yield meaningful scores in terms of diagnosis and prognosis regardless of the test taker's age and education [24]. It appears that education-corrected and uncorrected scores meet this criterion equally well.

Given that our participants were recruited from general hospitals our study might be limited with regard to the generalizability of results. Strengths of our study were the large number of participants and its prospective design, which allowed us to examine the effect of comparative norms on the predictive validity of neuropsychological test scores with high statistical power.

The notion that the application of age-norms attenuates the validity of test scores in reflecting the risk of dementia has been described within the context of MCI [21] and emphasized in cross-sectional studies with dementia as outcome variable [22]–[27]. The present study is the first to complement these findings with results from a prospective design directly related to MCI. Our findings provide evidence for the notion that applying age-norms in the diagnosis of MCI decreases the prognostic value of the concept by overestimating the risk of progression in younger cases and underestimating the risk in older cases [21]. In all likelihood, the omission of age-correction can improve the clinical value of MCI and lead to a more accurate identification of individuals at risk of dementia [20], [21]. Importantly, this notion should inform the refinement of diagnostic criteria of MCI and guide the operationalization of cognitive impairment in MCI in future conversion studies. As argued above, applying higher standards to older individuals than to younger individuals might actually increase the accuracy of risk estimation even more, since it takes the effects of brain reserve into account [29]. Even though this notion is reasonable from a theoretical point of view, its validity remains to be investigated.

The implications of education-correction of test scores have hardly been considered within the context of MCI, if at all. Based on the results of the present study and the established finding that higher education represents higher brain reserve [29]–[31], it can be argued that education-correction of test scores leads to a more accurate identification of individuals at risk of dementia. Individuals with higher education are expected to show better cognitive test performance than individuals with lower education in order to be not considered at increased risk. Norms based on educational level accommodate this actuality and, therefore, allow for a more accurate identification of individuals at risk of dementia. Because our study was the first to examine this question and differences between education-corrected and uncorrected scores were only small, further investigation in this area is necessary. Additional support for the above conclusions about age- and education-norms is given by the finding that education-correction reduced the influence of the possible confounders age and education the most and age-correction the least.

The results of the present study also have implications for MCI within the context of screening for dementia and its role in the early detection of individuals at risk. It has been suggested that MCI represents early-stage dementia [47], [48] which implies that progression is inevitable and MCI should, therefore, play a crucial part in early detection and intervention. However, partly due to its heterogeneous and potentially reversible etiology [49], most cases of MCI do not progress to dementia over the next years [14], [15]. Actually, a diagnosis of MCI can bear only little prognostic value and cause more harm than good [50]. On the other side of the same coin, results from a prospective study suggested that a considerable number of cases that were not included by several definitions of MCI progressed to dementia within two years [51]. Taken together, it appears that even though for a subgroup MCI represents an early sign of incipient dementia, current definitions of MCI and their implementation in research and practice are off the mark in capturing this subgroup. The common application of age-norms in the diagnostics of MCI likely contributes to this. Education-norms, however, which can increase predictive accuracy, are often neglected [1]. Our findings highlight that age and education are important sources of information that need to be properly employed to zero in on those at increased risk of dementia.

In conclusion, the results of the present study suggest that the application of age-norms decreases the validity of cognitive test scores in predicting progression to dementia within three years. In contrast, the application of an education-norm likely increases the predictive accuracy. As the detection of individuals at risk of dementia is the main value of MCI, these findings should be considered with regard to how cognitive impairment is operationalized and diagnosed in research and practice.
